# Autologous Peroneal Artery Interposition Graft for Traumatic Reconstruction of the Anterior Tibial Artery: A Case Report

**DOI:** 10.7759/cureus.117959

**Published:** 2026-10-15

**Authors:** Lahcen Kaddouri, Yassine Bachar, Youssef Belchiti, Youssef Benhmammouche, Kenza Benzmane, Abdelkarim Kharroubi

**Affiliations:** 1 Vascular Surgery, Centre Hospitalier Universitaire Mohammed VI, Agadir, MAR; 2 Vascular Surgery, Université Ibn Zohr, Agadir, MAR

**Keywords:** anterior tibial artery, autologous, limb salvage, peroneal artery, vascular system injuries

## Abstract

Autologous vein is the preferred conduit for many peripheral arterial reconstructions, but an inadequate caliber may limit its use. A 27-year-old man presented after a road traffic accident with an anterior tibial artery injury and associated muscular and nerve injuries. The ipsilateral great saphenous vein measured approximately 1.4 mm and was considered unsuitable. A healthy 5-cm segment of the middle peroneal artery was used as an interposition autograft. Distal perfusion improved immediately, with palpable dorsalis pedis and posterior tibial pulses. At three months, clinical perfusion remained satisfactory, neurological function had improved, and wound healing was progressing. Duplex ultrasonography demonstrated flow in the examined graft segment, although comprehensive hemodynamic data were unavailable. This case illustrates a selected application of arterial autografting when the available venous conduit is unsuitable. Longer follow-up is needed to assess durability.

## Introduction

Infrapopliteal arterial reconstruction is challenging because of small vessel caliber and associated soft-tissue injuries. When primary repair cannot be achieved without tension, autologous vein is generally preferred; however, inadequate vein caliber may limit its use [1-3].

Native arterial autografts offer an uncommon alternative in selected circumstances. Franz and Foster reported tibial reconstruction using a peroneal artery segment, with patency at one year [4]. We describe anterior tibial artery reconstruction using a 5-cm middle peroneal artery autograft after a road traffic accident when the available ipsilateral great saphenous vein was considered unsuitable. The case is presented using the CARE reporting framework [5].

## Case presentation

Patient information and clinical findings

A 27-year-old man was admitted approximately three hours after a road traffic accident with a deep traumatic wound of the right leg (Figure 1). On admission, he was conscious, pale, and diaphoretic, with a blood pressure of 85/50 mmHg, a heart rate of 120 beats/min, and a hemoglobin level of 8.2 g/dL. Initial resuscitation included transfusion of three units of packed red blood cells and hemodynamic stabilization. His respiratory status was stable. No associated fracture was identified.

**Figure 1:**
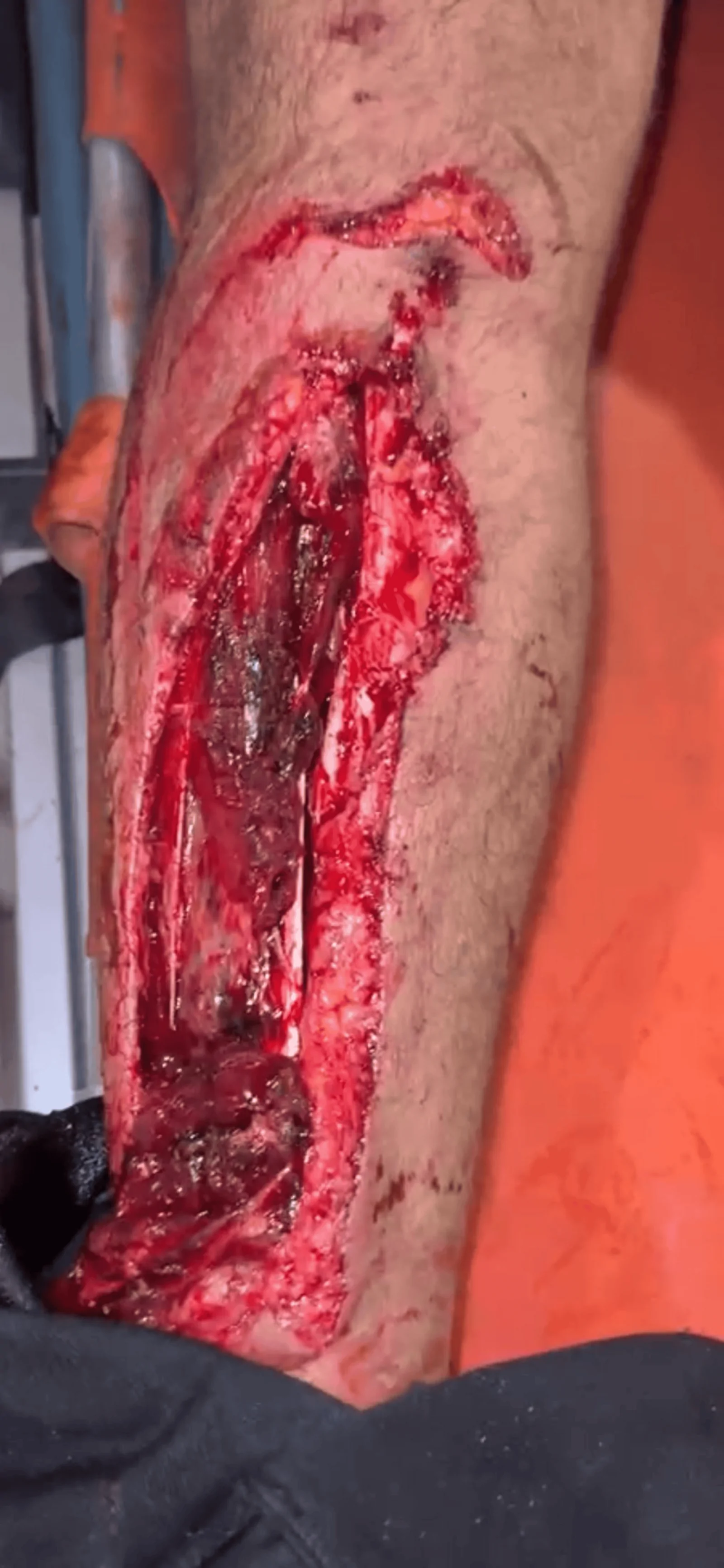
Initial appearance of the deep traumatic wound involving the anterolateral aspect of the leg, with associated soft-tissue injury.

The right foot was pale and cold, with prolonged capillary refill. The posterior tibial pulse was palpable, but the dorsalis pedis pulse was absent. Pallor, coldness, and prolonged capillary refill persisted after transfusion and hemodynamic stabilization. Neurological examination showed that ankle dorsiflexion and toe extension were both graded 1/5 on the Medical Research Council (MRC) scale, with reduced sensation in the first dorsal web space. These findings were consistent with deep fibular nerve involvement, although associated muscle injuries could also have contributed to the motor deficit. The open wound over the anterior tibial artery, absent dorsalis pedis pulse, and persistent impairment of distal perfusion prompted urgent operative exploration.

Diagnostic assessment and timeline

Given the patient’s hemodynamic instability and clinical findings strongly suggestive of arterial injury, he was transferred directly to the operating theater for urgent surgical exploration, hemostasis, and revascularization. No preoperative imaging or ankle-brachial index measurement was performed.

Admission occurred approximately three hours after the injury. Following resuscitation, urgent exploration and anterior tibial artery reconstruction were performed. Reperfusion was achieved approximately one hour after admission, corresponding to approximately four hours after the trauma. Immediate postoperative examination showed a warm foot, normal capillary refill, and palpable dorsalis pedis and posterior tibial pulses.

Operative management

Surgical exploration revealed an approximately 4-cm contused segment of the distal right anterior tibial artery, together with injuries to the tibialis anterior and extensor digitorum longus muscles and the deep fibular nerve. No associated tibial or fibular fracture was identified. The distal peroneal artery was severely lacerated and shredded, whereas its middle segment appeared macroscopically healthy.

Systemic heparin was administered at a dose of 50 IU/kg before arterial clamping, which was performed using atraumatic bulldog clamps. Following debridement and preparation of healthy proximal and distal arterial ends, the anterior tibial artery defect precluded a tension-free primary anastomosis. The ipsilateral great saphenous vein was assessed as a potential conduit but measured approximately 1.4 mm in diameter and was considered unsuitable because of its caliber mismatch with the recipient artery.

Before donor harvest, the posterior tibial pulse was reassessed intraoperatively and remained palpable. This clinical finding, together with the severe distal peroneal artery injury, informed the individualized decision to use its healthy middle segment as a conduit. A segment approximately 5 cm long and 2.5 mm in diameter was harvested. Both the proximal and distal donor-artery ends were ligated, sacrificing peroneal arterial continuity. The preharvest assessment described in this report was clinical; it did not provide a comprehensive assessment of the remaining foot circulation.

Vascular flushing was performed with heparinized saline, and the graft was positioned between the prepared ends of the anterior tibial artery without tension, torsion, or kinking. Proximal and distal end-to-end anastomoses were completed using 6-0 double-armed polypropylene sutures, with careful intimal apposition and preservation of the luminal diameter (Figure 2).

**Figure 2:**
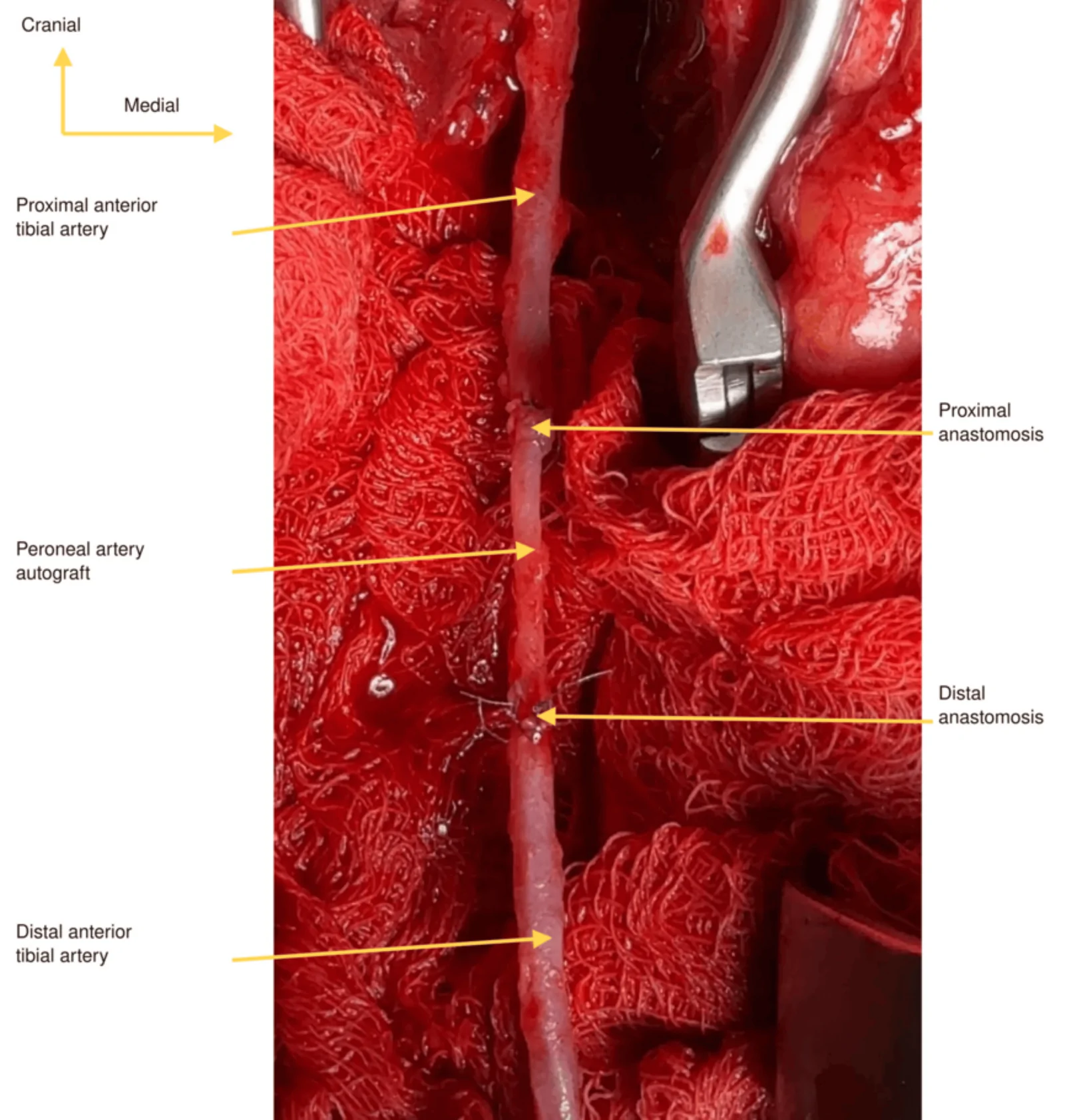
Intraoperative view of anterior tibial artery reconstruction using an autologous peroneal artery interposition graft. The arterial autograft is positioned between the proximal and distal ends of the anterior tibial artery with two end-to-end anastomoses.

After completion of the distal anastomosis, the clamps were released. Both the dorsalis pedis and posterior tibial pulses were palpable, the foot became warm, and capillary refill normalized. These findings documented immediate clinical reperfusion after reconstruction. The injuries to the tibialis anterior and extensor digitorum longus muscles and the deep fibular nerve were repaired by the orthopedic trauma team during the same operation.

No fasciotomy was performed. The wound was closed primarily without the need for flap coverage. Postoperative wound care consisted of daily cleansing with saline and application of an Urgosorb dressing.

Postoperative course and follow-up

The immediate postoperative course was favorable. Postoperative antiplatelet therapy consisted of aspirin at a dose of 75 mg once daily, prescribed for three months. Serial clinical examinations showed a warm, well-perfused foot, a palpable dorsalis pedis pulse, and normal capillary refill. No clinical signs of recurrent ischemia or other immediate vascular complications were observed. The patient was discharged after a seven-day hospital stay.

At the three-month follow-up, the right foot remained warm, capillary refill was normal, and the dorsalis pedis pulse was palpable. Sensation in the first dorsal web space had improved. Following physiotherapy, ankle dorsiflexion and toe extension had both improved from MRC grade 1/5 at admission to 4/5, indicating persistent mild weakness. Rehabilitation focused on muscle strengthening, ankle mobility, gait, and functional recovery. Despite primary closure at the initial operation, wound healing remained incomplete at three months, with limited superficial areas that had not fully epithelialized and required continued local wound care (Figure 3).

**Figure 3:**
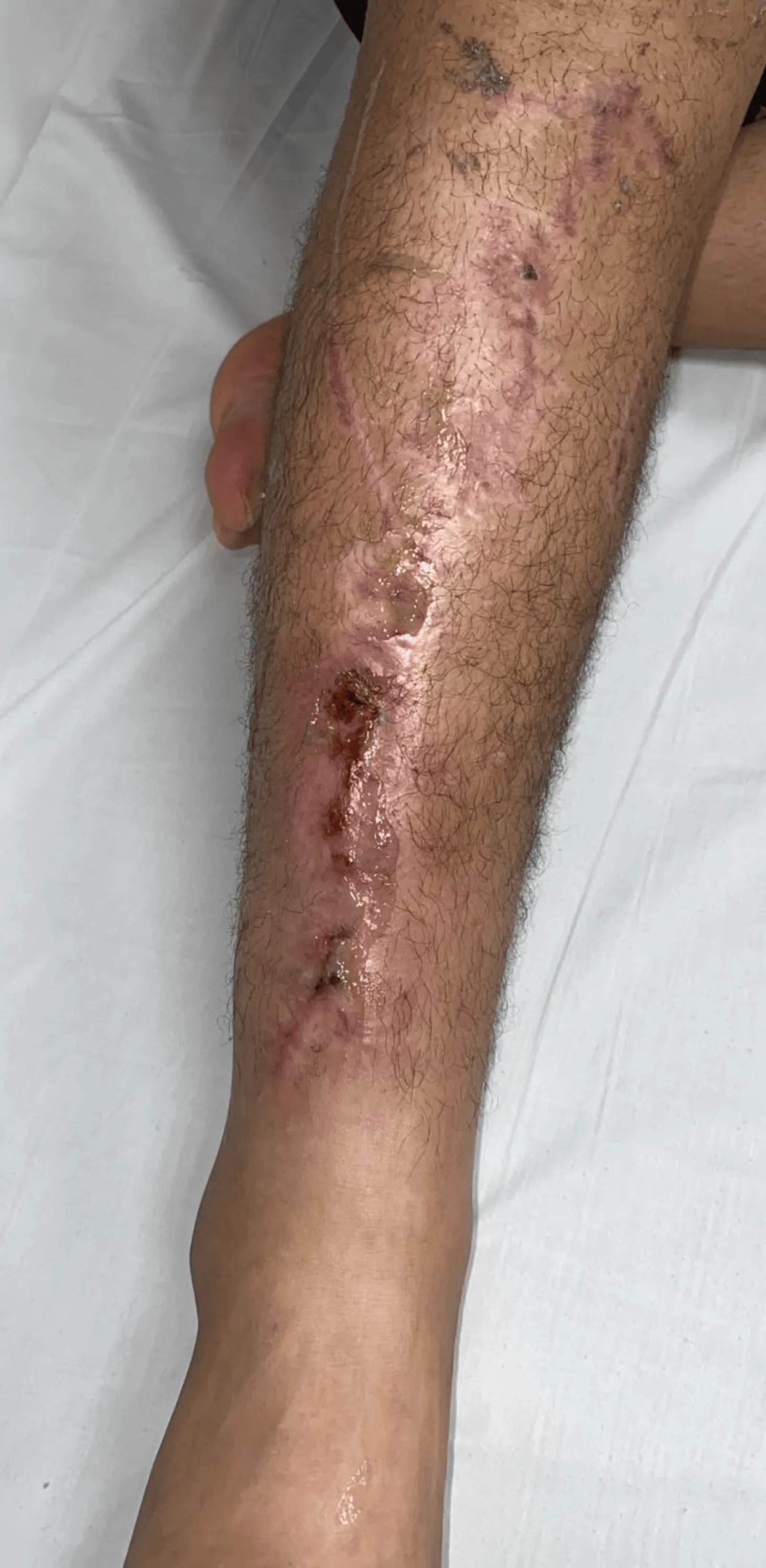
Clinical appearance of the affected leg at three months showing ongoing wound healing with limited residual superficial areas.

Duplex ultrasonography was the only postoperative vascular imaging examination performed. The available recording demonstrated flow in the examined graft segment; however, a complete segment-by-segment hemodynamic assessment was not available (Figure 4). No additional postoperative angiographic or cross-sectional vascular imaging was performed, as the clinical course remained favorable, with preserved limb perfusion and no clinical signs of recurrent ischemia.

**Figure 4:**
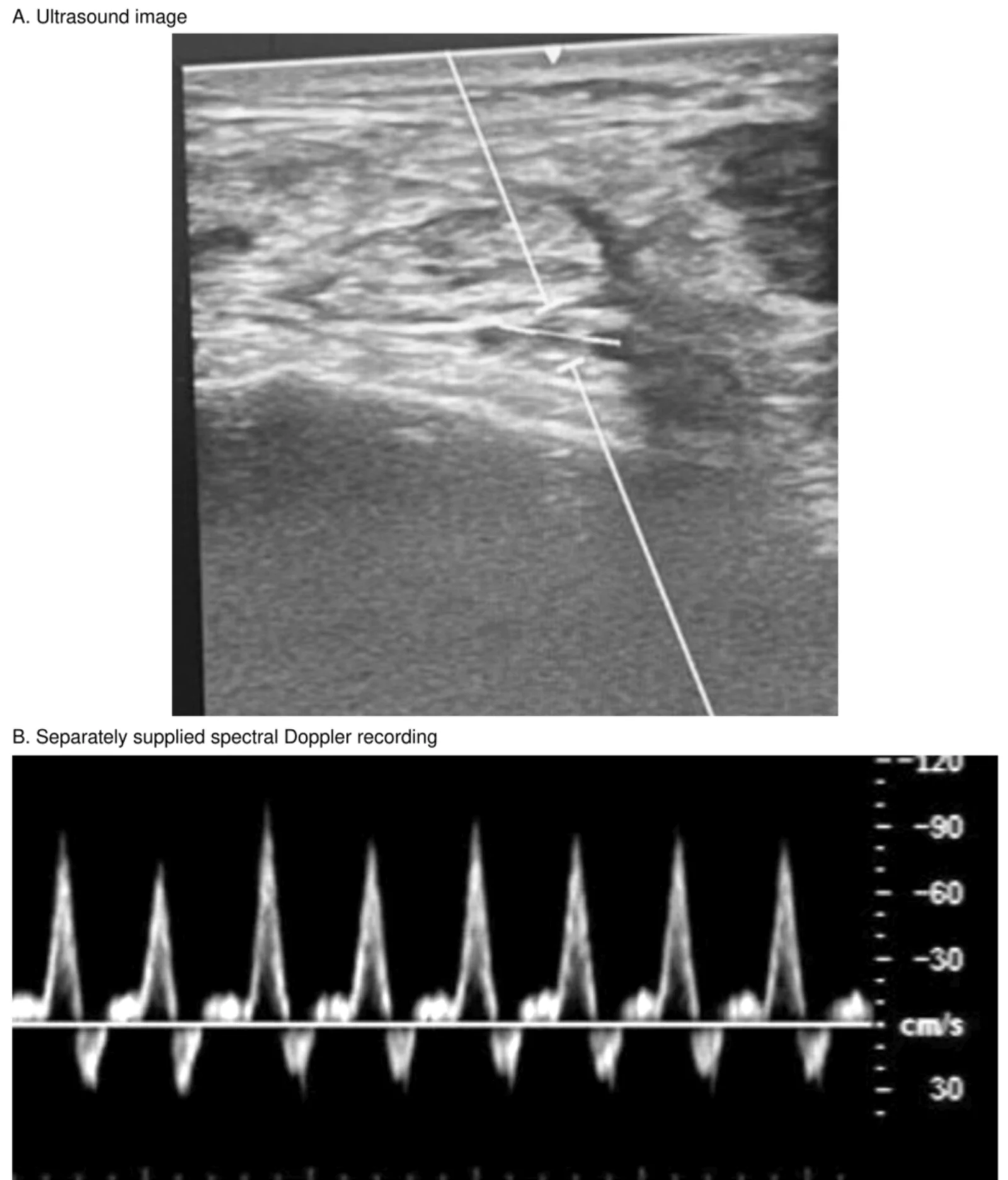
Duplex ultrasound recording at the three-month follow-up demonstrating flow in the examined arterial autograft segment.

## Discussion

This case illustrates the use of a peroneal artery autograft for a traumatic anterior tibial artery defect when the available ipsilateral great saphenous vein was unsuitable because of its small caliber. An approximately 4-cm contused anterior tibial segment required debridement, preventing a tension-free primary anastomosis. A macroscopically healthy 5-cm middle peroneal segment offered a closely matched conduit. The observed findings support feasibility in this individual case, without establishing superiority over venous reconstruction or the general safety of donor-artery sacrifice.

The indication for reconstruction deserves consideration because a posterior tibial pulse was palpable. Systemic shock could have contributed to the initial perfusion abnormalities. However, the foot remained pale and cold, with prolonged capillary refill after transfusion and hemodynamic stabilization, and the dorsalis pedis pulse remained absent. These persistent clinical findings and the confirmed anterior tibial lesion supported the individualized decision to restore anterior tibial continuity. This case should not be interpreted as evidence that every isolated anterior tibial injury requires reconstruction when adequate foot perfusion is otherwise maintained.

Autologous vein remains the reference conduit for most small peripheral arterial reconstructions. The 2025 European Society for Vascular Surgery guidelines advise consideration of vein grafts for small-caliber distal vessels or repairs requiring longer conduits [1]. Brazilian vascular-trauma guidelines similarly recommend autologous vein for most limb vessels and prefer it for vessels 7 mm or smaller [2]. These recommendations reflect infection resistance, handling characteristics, and accumulated clinical experience. The present case deviated from the usual approach because the available saphenous vein had a pronounced caliber mismatch. This decision should be described as individualized rather than as evidence of the superiority of an arterial autograft.

An autologous arterial segment offers theoretical advantages, including close caliber matching, a wall adapted to arterial pressure, and avoidance of prosthetic material. These advantages must be balanced against donor-site ischemia, loss of an arterial runoff vessel, spasm, and uncertain long-term behavior. Infrapopliteal circulation involves the anterior tibial, posterior tibial, and peroneal arteries; the peroneal artery may contribute to foot perfusion through its distal branches and collateral connections. Its sacrifice therefore requires careful assessment of the remaining arterial supply.

In this patient, the distal peroneal artery was severely shredded, and the posterior tibial pulse was palpable both at admission and intraoperatively before harvest. Both donor ends were ligated after segment removal. These findings explain the operative choice but do not quantify residual peroneal runoff or establish that the injured vessel had no useful contribution to foot perfusion. Preharvest assessment relied on clinical palpation rather than a documented comprehensive hemodynamic evaluation. Postreconstruction pulses and improved perfusion describe the outcome; they cannot retrospectively establish the adequacy of the preharvest assessment.

Franz and Foster described an approximately 4-cm proximal peroneal artery autograft connecting the tibioperoneal trunk to the posterior tibial artery after a gunshot injury, with patency documented at one year [4]. Our case illustrates a different application: a 5-cm middle peroneal segment used for anterior tibial artery interposition after a road traffic accident. Whereas their conduit selection was motivated partly by the need to expedite reconstruction, ours was driven by the small caliber of the available ipsilateral great saphenous vein and the availability of a healthy arterial segment. This adds to the limited clinical experience with peroneal artery autografts without establishing superiority over venous reconstruction.

Technical execution is central in small-vessel reconstruction. Adequate debridement to healthy intima, a tension-free configuration, atraumatic handling, precise intimal apposition, avoidance of narrowing, and confirmation of inflow and distal runoff are established principles [1-3]. The immediate return of a pedal pulse, warmth, and normal capillary refill supported early technical success. At three months, the available duplex recording demonstrated flow in the examined graft segment.

This report has several limitations. A single case cannot establish comparative effectiveness or support routine use of this technique. Donor-vessel selection was individualized, and the clinical preharvest assessment did not comprehensively characterize residual foot perfusion or the contribution of the injured peroneal artery. Follow-up was limited to three months. Although motor strength improved from MRC grade 1/5 to 4/5, residual weakness and incomplete wound epithelialization persisted. Duplex documentation demonstrated flow in the examined segment but did not provide a complete evaluation of the graft, both anastomoses, and the remaining arterial axes.

## Conclusions

In this individual case, a healthy middle peroneal artery segment was used to reconstruct a traumatic anterior tibial artery defect when the available ipsilateral venous conduit was unsuitable. Immediate clinical reperfusion was observed, motor strength improved during rehabilitation, and duplex ultrasonography demonstrated flow in the examined graft segment at three months. These observations support feasibility in a selected patient but do not establish complete graft patency, long-term durability, or the general safety of peroneal artery sacrifice.
